# Supplementary material for: BMP2 Diminishes Angiotensin II-Induced Atrial Fibrillation by Inhibiting NLRP3 Inflammasome Signaling in Atrial Fibroblasts
Source: Biomolecules. 2024 Aug 25;14(9):1053. doi: 10.3390/biom14091053 (PMC11430365; doi:10.3390/biom14091053)
Supplement: Supplementary file 1 [file biomolecules-14-01053-s001.zip › biomolecules-3116137-supplementary-revised .pdf]

Supplemental Material

Supplemental Figure S1

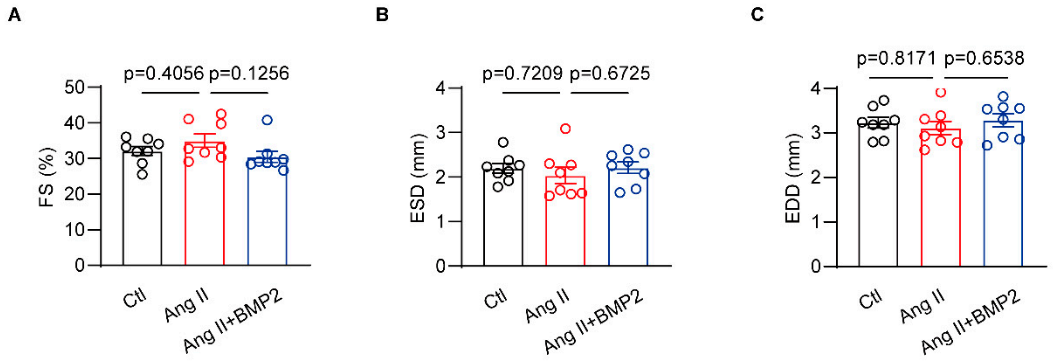

Supplemental Figure S1. Cardiac ventricle diameter in three groups of rats

(**A**) The measurements of left ventricular fraction shortening of Ctl, Ang II and Ang II+BMP2 rats by echocardiography, n=8/group. (**B** and **C**) The measurements of ventricle systolic and diastolic diameters in Ctl, Ang II and Ang II+BMP2 rats by cardiography, n=8/group. The bar graph data are mean±SEM with individual values. Ctl, control; angiotensin II, Ang II; FS, fraction shortening; ESD, end-systolic diameter; EDD, end-diastolic diameter. p-values are determined with one-way ANOVA and Turkey's multiple comparisons test in **A-C**.

Supplemental Figure S2

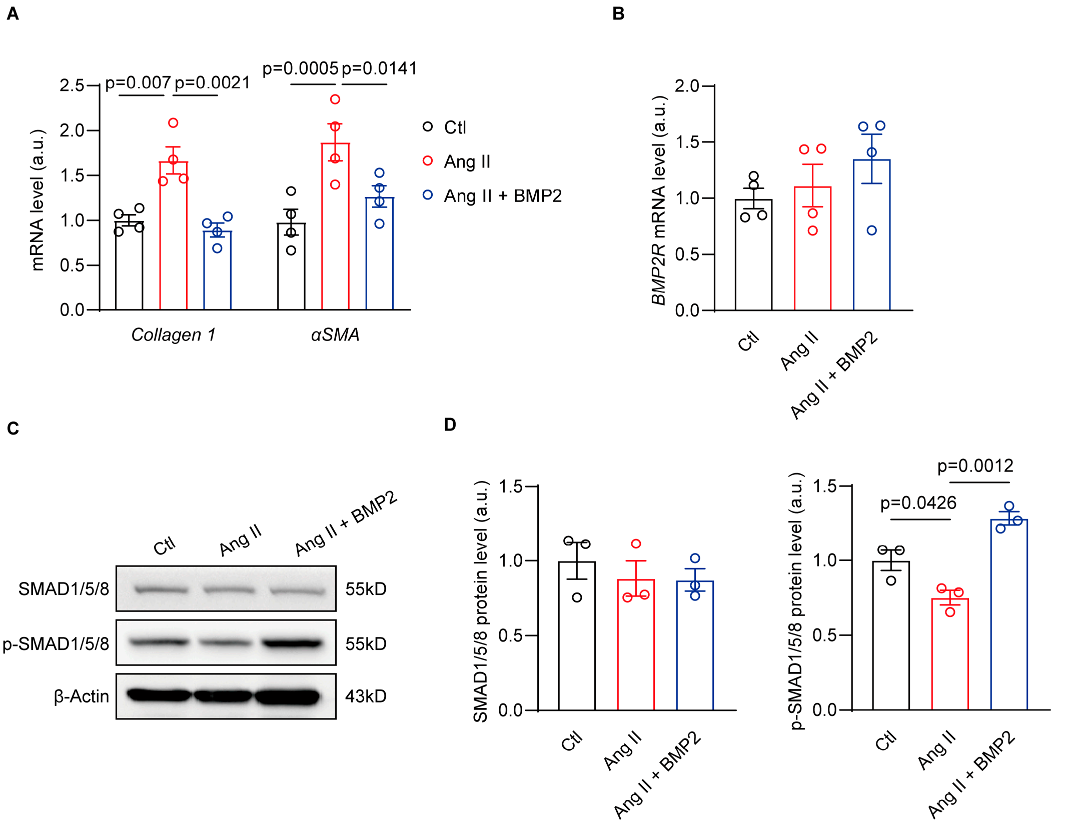

Supplemental Figure S2. Fibrosis marker expressions in rat atria.

(**A**) The mRNA levels of *Collagen1* and *αSMA* of Ctl, Ang II and Ang II+BMP2 rat atria by qRT-PCR assay, n=4/group. (**B**) The expression of *BMP2R* mRNA in Ctl, Ang II and Ang II+BMP2 rat atria by qRT-PCR assay, n=4/group. (**C** and **D**) The expressions of SMAD1/5/8 and p-SMAD1/5/8 preotein in Ctl, Ang II and Ang II+BMP2 rat atria by western blot, n=3/group. The bar graph data are mean±SEM with individual values. Ctl, control; angiotensin II, Ang II. p-values are determined with two-way ANOVA and Turkey's multiple comparisons test in **A;** p-values are determined with one-way ANOVA and Turkey's multiple comparisons test in **B** and **D**.

Supplemental Figure S3

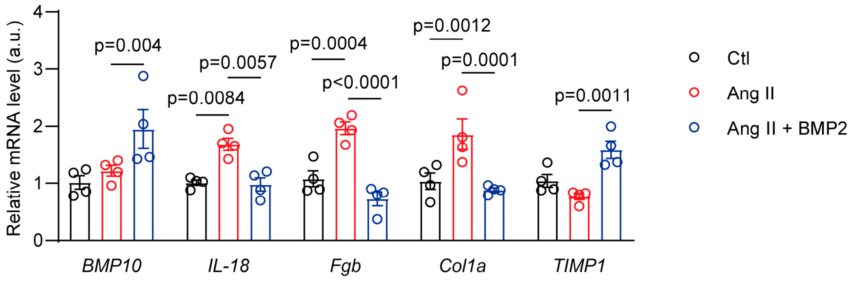

Supplemental Figure S3. qRT-PCR assay to verify key gene expressions in rat atria.

The mRNA levels of *BMP10, IL-18, Fgb, Col1a* and *TIMP1* of Ctl, Ang II and Ang II+BMP2 rat atria by qRT-PCR assay, n=4/group. The bar graph data are mean±SEM with individual values. Ctl, control; angiotensin II, Ang II. p-values are determined with two-way ANOVA and Turkey's multiple comparisons test in the figure.

Supplemental Table S1. Basic clinical information of enrolled individuals.

Characteristics	SR group (n=6)	AF group (n=6)	p value
Age	64.0±2.73	62.3±2.99	0.6892
Gender, n (%)			0.5671
Male	2 (33.33)	4 (66.67)	
Female	4 (66.67)	2 (33.33)	
BMI (kg/m^2)	25.35±0.87	23.9±1.16	0.3425
Smoking, n (%)	3 (50.00)	2 (33.33)	>0.9999
Hypertension, n (%)	3 (50.00)	3 (50.00)	>0.9999
Diabetes, n (%)	1 (16.67)	2 (33.33)	>0.9999
CHD, n (%)	4 (66.67)	2 (33.33)	0.5671
HF, n (%)	1 (16.67))	3 (50.00)	>0.5455
CKD, n (%)	1 (16.67)	1 (16.67)	>0.9999
Cr (mmol/L)	67.9±7.27	82.9±6.64	0.1599
UA (mmol/L)	358.9±36.89	420.1±48.47	0.3386
NT-proBNP (pg/L)	251.5±99.01	411.7±145.2	0.3834
LAD (mm)	36.5±2.41	44.67±1.50	0.0163
RAD (mm)	45.17±2.17	52.33±3.15	0.0902
LVEF (%)	57.67±3.43	56.50±4.24	0.8350

BMI, body mass index; CHD, Coronary heart disease; CKD, chronic kidney disease; Cr, creatinine; HF, Heart Failure; LAD, left atrial diameter; LVEF, left ventricle ejection fraction; NT-proBNP, N-terminal pro-brain natriuretic peptide; RAD, right atrial diameter; UA, uric acid. (*p<0.05, ** p<0.01, *** p<0.001, **** p<0.0001)

Supplemental Table S2. ECG of rats in Figure 2.

	Ctl (n=8)	Ang II (n=8)	Ang II+BMP2 (n=8)
HR (bpm)	448.0 ± 11.52	451.6 ± 11.70	449.8 ± 10.46
P wave (ms)	23.4 ± 0.48	22.85 ± 0.68	23.44 ± 0.43
RR (ms)	141.4 ± 4.90	140.1 ± 6.35	142.3 ± 4.95
PR (ms)	45.1 ± 0.55	45.8 ± 0.67	45.7 ± 0.52
QRS (ms)	17.9 ± 0.38	17.1 ± 0.43	16.52 ± 0.35
QT (ms)	71.8 ± 0.45	71.3 ± 0.52	71.1 ± 0.44

HR, heart rhythm; RR, R-R interval; PR, P-R interval; QRS, QRS duration; QT, QT interval.

Supplemental Table S3. Differential genes by RNA sequencing in Figure 3.

gene	log2 (Fold_change)	p-value
Rn7sl1	-1.86055	2.27E-06
Col1	-1.51888	3.49E-06
Il18	-2.25884	6.09E-06
Fgb	-2.28382	5.58E-05
Hpx	-2.01499	5.82E-05
F2	-2.49834	0.001292
Timp1	0.874923	0.002326
Aldob	-2.10552	0.003152
Cpa6	-0.99245	0.003605
Upp1	-0.82887	0.003681
Plin5	-0.95465	0.003971
LOC102548668	-2.34361	0.004557
Bmp10	0.852342	0.004635
Scd2	0.74347	0.004646
LOC108349236	-1.0249	0.004726
RGD1564801	-1.49432	0.004911
Lrrc2	-1.65129	0.005548
St8sia5	0.962669	0.005674
LOC108351712	-2.0209	0.005766
Fras1	0.884953	0.005947
Gkn1	-2.84756	0.006807
Slc17a7	0.896791	0.007051
Cpt1a	-1.42355	0.007522
Mat1a	-1.88944	0.008001
Ogn_ID:291015	1.266309	0.0081
Cadps	-0.99886	0.008223
LOC315661	-2.29281	0.008538
LOC108352946	-1.32714	0.008785
LOC108349780	3.42042	0.008861
Tmem140	-0.76263	0.009724
Serpinc1	-1.98717	0.009792
Itih1	-2.92875	0.010629
Cyp2e1	-3.95461	0.010695
LOC100360522	1.065376	0.010707
Uox	-2.16141	0.012575
Acot7	-0.71477	0.012774
Grap2	-1.75514	0.01322
Srebf1	0.654306	0.01354
LOC100362895	-0.66552	0.013902
LOC102546455	-0.47515	0.014991

LOC100360791	1.895056	0.015054
LOC108352934	-4.46423	0.015367
RGD1312005	-0.99065	0.017053
Ccdc147	-1.23065	0.018659
Dsg3	-3.475	0.018846
Tdo2	-1.59683	0.018966
LOC103693985	-2.07306	0.018996
Ddn	1.067423	0.019774
Hsdl2	-1.23207	0.019965
Susd4	0.860065	0.020125
Kif27	-2.72082	0.020164
Ces4a	2.678978	0.020979
Gckr	-1.87019	0.021038
Tspan10	0.57012	0.02148
LOC108353074	-1.27019	0.021684
Snap25	-0.76941	0.022874
Carns1	-0.93827	0.02313
LOC310926	0.630216	0.023642
LOC102550095	-2.89475	0.024577
Mcemp1	0.980216	0.025643
Slc25a20	-0.69741	0.025862
Ybx1-ps3	1.039781	0.026166
Otc	-1.88196	0.026264
Nol8_ID:361221	3.106879	0.027148
Mrpl1	-0.97695	0.027674
Sbspon	0.707831	0.027821
Cdh12	-1.74611	0.028361
Kndc1	-1.84441	0.028378
RGD1564247	1.739888	0.028635
Eci2	-0.60707	0.029082
LOC108351905	1.34963	0.029594
LOC102550607	-1.30507	0.029774
Plet1	-2.06532	0.029989
LOC108348621	1.874239	0.030853
LOC102550725	-3.83386	0.03127
LOC108349238	1.03576	0.031762
LOC108348047	-2.5153	0.031788
Bbox1	-2.68986	0.032378
Thbs4	-1.64397	0.032598
LOC108352829	1.760637	0.033851
LOC102547398	3.064049	0.034265
LOC100910771	1.64945	0.034605

Syt4	-0.86887	0.035135
Cp	-0.51771	0.035472
Sh3rf3	0.691128	0.035603
LOC100910366	-1.516	0.035729
LOC102554253	0.697887	0.035981
Rnf207	0.549474	0.036125
ENSRNOG00000052332.1	0.9871	0.036308
Ano5	-0.80483	0.036783
Zwint	-0.71513	0.036884
Slc25a34	0.76904	0.03711
Lrrd1	-1.45613	0.03777
LOC102550010	-2.01722	0.038032
LOC102553541	-0.72985	0.038203
Cps1	2.203876	0.038266
LOC103692417	3.086698	0.038296
Gprasp1	-0.72061	0.038591
Cyp4v3	-0.75929	0.03923
Lrrn1	1.655476	0.039346
Asmtl	0.571835	0.039352
Dcst1	2.995898	0.039762
Clec1b	1.517039	0.039799
Prr32	2.085561	0.040044
Ghrhr	0.36974	0.040136
Th	-2.4296	0.040416
LOC102551701	-1.0369	0.04065
Mt2A	-0.59002	0.040819
Slc2a3	-0.9832	0.040888
Frmpd4	-1.16605	0.041489
Smtnl1	-2.21232	0.04175
ENSRNOG00000051226.1	-2.27135	0.041882
Nxph4	-3.23068	0.041936
Kcnk9	-2.66867	0.042007
Spata17	-2.66439	0.042053
LOC108350411	-3.0157	0.042107
Slc25a29	0.814686	0.042238
ENSRNOG00000059125.1	-2.33125	0.042431
LOC102551687	-3.40641	0.042686
Luzp2	-1.79018	0.042746
Ucma	-1.34415	0.042976
Icoslg	-0.59362	0.043047
Snrpa	0.789464	0.043132
Gjb1	-2.23058	0.043809

Ifitm3	-0.61668	0.044408
LOC681184	2.645612	0.04461
Cacna1h	0.614779	0.044841
Cyp1b1	-0.49543	0.044926
LOC102556878	1.544896	0.045015
LOC102555574	1.568155	0.045407
LOC100909776	0.681367	0.045453
Chrna7	-0.76289	0.045589
LOC100909441	1.233357	0.04581
Rn18s	-0.7329	0.045847
Slc9b2	-1.53671	0.045899
Slc29a4	-1.18094	0.045916
Cilp2	-2.61171	0.046352
LOC102548893	2.460965	0.046857
Mas1l	0.813668	0.046952
LOC100360508	1.01598	0.047521
Mtus2	-0.53875	0.047654
Avil	-1.40007	0.047698
LOC108349243	3.382524	0.048449
Acot4	-4.40794	0.048569
Trem2	-1.03575	0.049212
Gnb3	-0.5987	0.049685
Pde4c	1.342909	0.049976

Western blot antibody

BMP2, Abcam, Rabbit, ab284387;

SMA, Abcam, Mouse, ab7817;

SMAD1/5/8, Abcam, Rabbit, ab13723;

p-SMAD1/5/8, CST, Rabbit, #13820;

GAPDH, CST, Rabbit, #2118.

qRT-PCR primers

BMP2, Human, Forward, TGTATCGCAGGCACTCAGGTCA

BMP2, Human, Reverse, CCACTCGTTTCTGGTAGTTCTTC

BMP2R, Human, Forward, AGAGACCCAAGTTCCCAGAAGC

BMP2R, Human, Reverse, CCTTTCCTCAGCACACTGTGCA

GAPDH, Human, Forward, GTCTCCTCTGACTTCAACAGCG

GAPDH, Human, Reverse, ACCACCCTGTTGCTGTAGCCAA

BMP2, Rabbit, Forward, TACACCGTGCGCAGCTTCCATC

BMP2, Rabbit, Reverse, CCGGAAGATCTGGAGTTCTGCAG

BMP2R, Rabbit, Forward, AGAGACCCAAGTTCCCAGAAG

BMP2R, Rabbit, Reverse, TCTCCTCAGCACACTGTGCAG

GAPDH, Rabbit, Forward, GTCTCCTCTGACTTCAACAGC

GAPDH, Rabbit, Reverse, ACCACCCTGTTGCTGTAGCCA

BMP2, Rat, Forward, AACACCGTGCGCAGCTTCCATC

BMP2, Rat, Reverse, CGGAAGATCTGGAGTTCTGCAG

GAPDH, Rat, Forward, GCAAGAGAGAGGCCCTCAG

GAPDH, Rat, Reverse, TGTGAGGGAGATGCTCAGTG

NLRP3, Rat, Forward, CAGACCTCCAAGACCACGACTG

NLRP3, Rat, Reverse, CATCCGCAGCCAATGAACAGAG

Caspase 1, Rat, Forward, TGCCTGGTCTTGTGACTTGGAG

Caspase 1, Rat, Reverse, ATGTCCTGGGAAGAGGTAGAAACG

FL-GSDMD, Rat, Forward, GGTGCTTGACTCTGGAGAACTG

FL-GSDMD, Rat, Reverse, GCTGCTTTGACAGCACCGTTGT

NT-GSDMD, Rat, Forward, ACCTGCTTTGACAGCACCGTTGT

NT-GSDMD, Rat, Reverse, TGTGCTTTGACAGCACCGTTGT

ASC, Rat, Forward, TTATGGAAGAGTCTGGAGCTGTGG

ASC, Rat, Reverse, AATGAGTGCTTGCCTGTGTTGG

Collagen1, Rat, Forward, CCTCAGGGTATTGCTGGACAAC

Collagen1, Rat, Reverse, CAGAAGGACCTTGTTTGCCAGG

αSMA, Rat, Forward, TGCTGACAGAGGCACCACTGAA

αSMA, Rat, Forward, CAGTTGTACGTCCAGAGGCATAG

Abbreviations

ACFs	atrial cardiac fibroblasts
AF	atrial fibrillation
Ang II	angiotensin II
BMP2	bone morphogenic protein 2
CFs	cardiac fibroblasts
EDD	end-diastolic diameter
ESD	end-systolic diameter
GO	gene ontology
GSDMD	gasdermin D
IL-1β	Interleukin 1β
IL-6	Interleukin 6
NC	Negative control
NLRP3	NOD-, LRR- and pyrin domain-containing protein 3
PES	programmed intracardiac stimulation
RAP	right atria tachypacing
ROS	reactive oxygen species
SR	sinus rhythm
TGF-β	transforming growth factor β
