# Supplementary figures and images for: BMP2 Diminishes Angiotensin II-Induced Atrial Fibrillation by Inhibiting NLRP3 Inflammasome Signaling in Atrial Fibroblasts
Source: Biomolecules. 2024 Aug 25;14(9):1053. doi: 10.3390/biom14091053 (PMC11430365; doi:10.3390/biom14091053)

Human atria (Figure 1B)

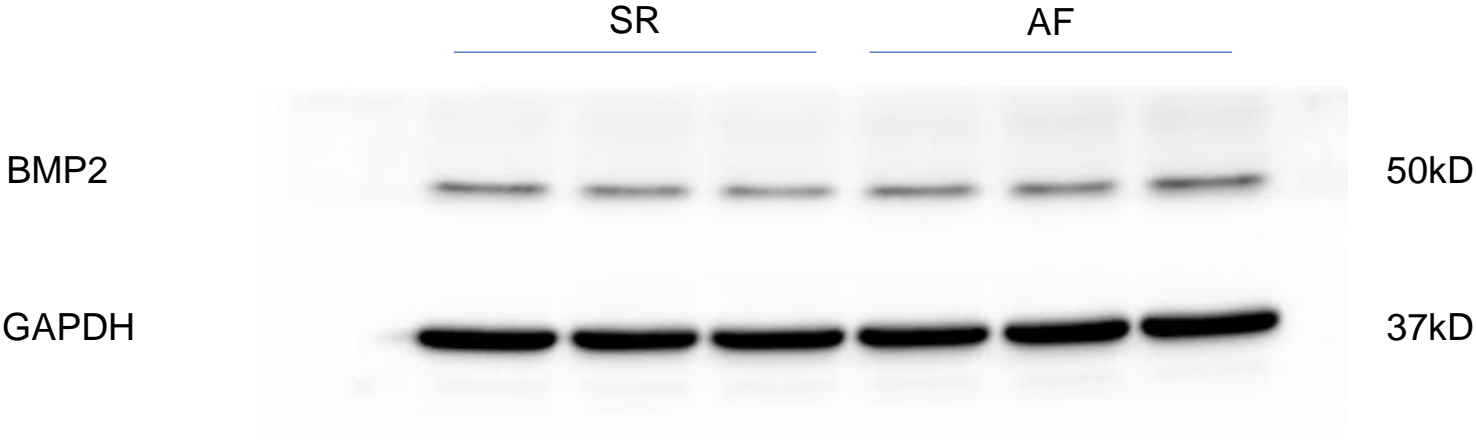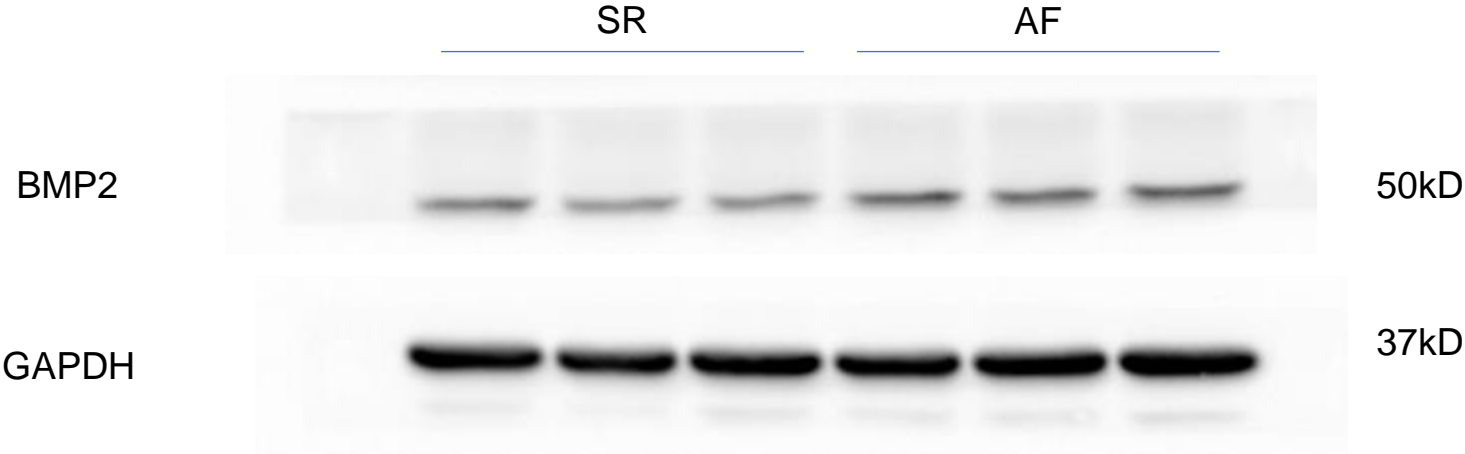

Rabbit atria (Figure 1E)

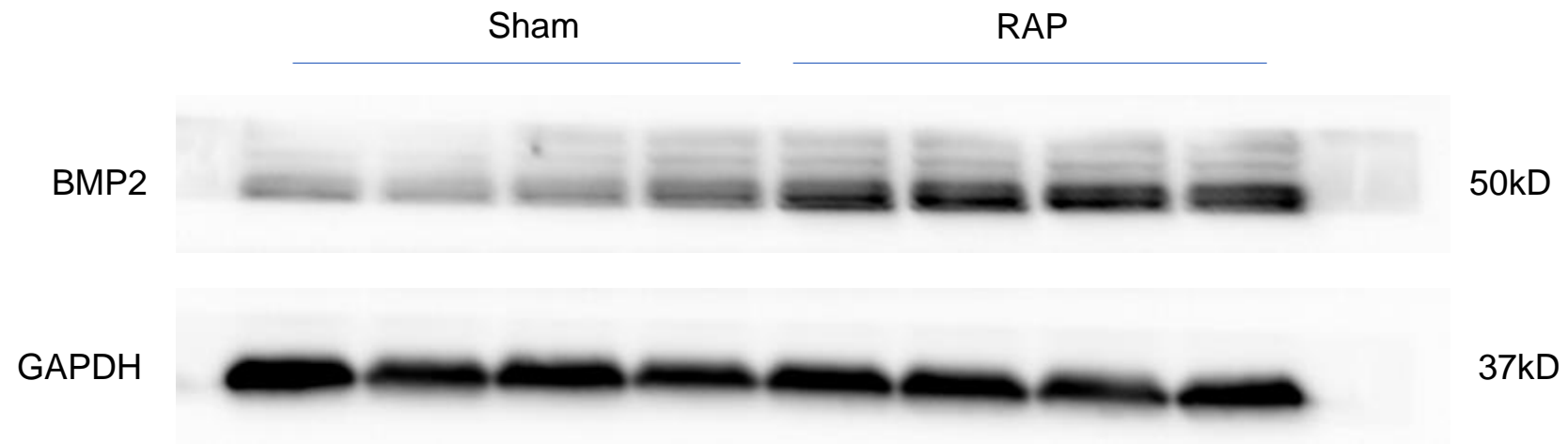

Rat atria (Supplemental Figure 2C)

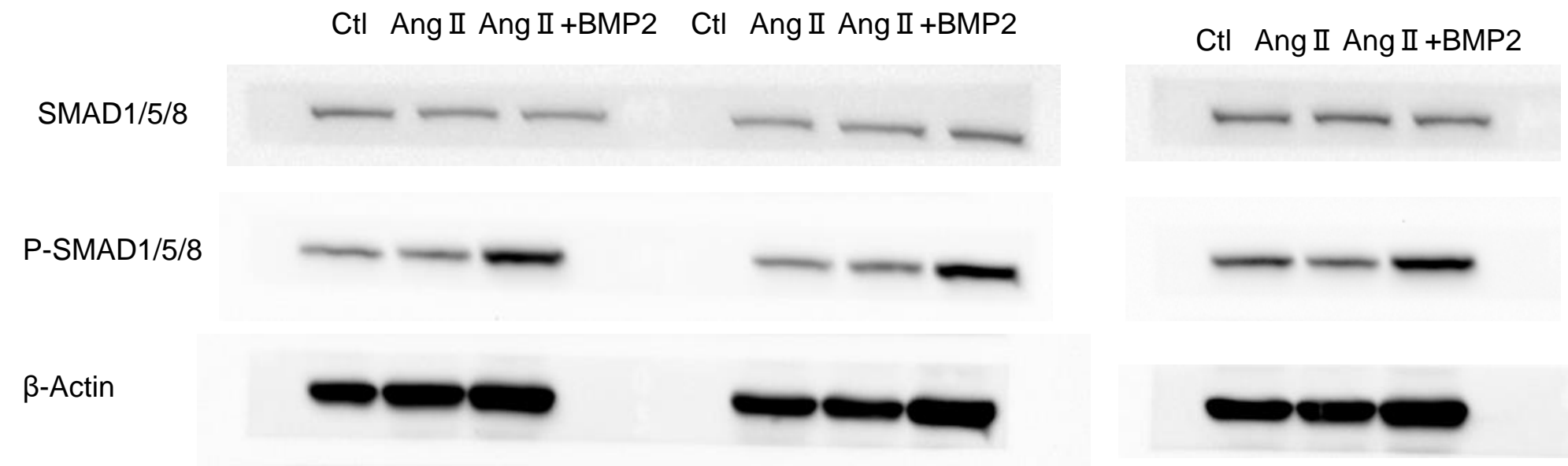

Supplement: Supplementary file 1 [file biomolecules-14-01053-s001.zip › original western blot.pdf]
